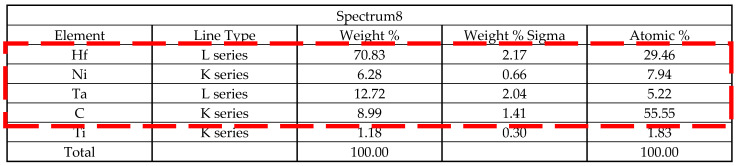# Influence of Hf and MC Carbide on Transverse Platform in Single Crystal Grain Selection of 2D Grain Selector

**DOI:** 10.3390/ma15186274

**Published:** 2022-09-09

**Authors:** Xintao Zhu, Fu Wang, Dexin Ma, Andreas Bührig-Polaczek

**Affiliations:** 1 Foundry Institute, RWTH Aachen University, Intzestrasse 5, 52072 Aachen, Germany; 2 Taizhou Jinying Precision CAST Co., Ltd., Taizhou 225714, China; 3 State Key Laboratory for Manufacturing System Engineering, School of Mechanical Engineering, Xi’an Jiaotong University, Xi’an 710049, China

**Keywords:** grain boundary single crystal, directional solidification, CM247LC, carbide precipitation

## Abstract

CM247LC Ni-based components have been widely used in developing hot ends in aero-engines and gas industrial turbines, and these have exhibited promising directional solidification (DS) results. However, the superalloy CM247LC shows defects after adding carbon (C) and hafnium (Hf). In this study, the effects of adding C and Hf on grain selection have been explored to enhance the 2D grain selector’s performance and reduce casting costs. The experimental results reveal that the final region of carbide formation is where the dendrite is pushed into the paste region and finally solidifies. The performance requirements of carbide on the alloy can be controlled by changing the paste region and solidification sequence.

## 1. Introduction

CM247LC Ni-based components are widely used in the hot ends of aero-engines and gas industrial turbines (GITs) because of their excellent performance at high-temperature [1]. However, it is observed that the addition of carbon (C) and hafnium in superalloy CM247LC could produce defects [2].

At present, the most common defect is the formation of carbide (MC) because it could affect the internal structure of a single crystal blade [3,4]. The presence of too many carbonized particles could cause crack nucleation and weakening of the mechanical properties of the superalloy. Carbide (MC) with a high melting point exists alone or in crystallized form and has a different structural orientation than the matrix structure. Therefore, carbide (MC) can be regarded as refined hybrid grains in superalloys [5]. The presence of carbide (MC) could serve as the crack’s initiation point so that it could play a vital role in the final fracture of a component.

Experimental data analysis reveals that carbide (MC) forms initially at the dendritic tip, followed by the initiation of γ/γ′-eutectics reaction on the carbide platform. In the γ/γ′-eutectics reaction, carbide (MC) serves as the core of hetero-nucleation, and floccule is precipitated at the final interface of solidification [3,6,7]. The results of EDS and SEM showed that the main component of floc was Hf, with a content concentration of 29.46%. Therefore, it is reasonable to conclude that Hafnium carbide, which is difficult to dissolve at high temperatures, is deemed to be the primary cause of floc production, and this floc eventually appears at the grain boundary. 

On the other hand, carbide precipitation at large-angle grain boundaries has a physical grain boundary-strengthening effect because carbides of the right size and shape become entangled with the dendrites on both sides of the grain boundary [8,9].

The strengthening mechanism is described below to understand better carbide’s beneficial effects: dislocation is the carrier of plastic movement of materials, and the stress field around solute atoms functions in conjunction with dislocation to create solid solution strengthening. A significant number of dislocations interact to form dislocation locks, dislocation blocks, or tangles, which inhibit their movement and result in deformation strengthening. Additionally, the precipitated phase interacts with dislocation, which leads to an increase in intensity [10]. Grain boundary strengthening is caused by the different orientations of grains on both sides of the grain boundary, the chaotic arrangement of atoms at the grain boundary, and the presence of other imperfections and impurity atoms so that the dislocation cannot continue to move and plug the grain boundary to achieve an enhancement effect. In addition, carbides at grain boundary generate dislocation locks, dislocation blocks, or tangles, which impede their movement and cause enhancement in deformation strengthening [9,11].

The effects of carbon and hafnium in CM247LC have not been systematically studied. This study focuses on the selection of grains in a 2D grain selector and depicts the evolution of the microstructure and grain growth in the 2D selector. The process of transverse platform grain texture evolution has been investigated using directional tests. Furthermore, the dendritic growth at the transverse platform of the contending section of the superalloy dendrites and the carbide precipitation process at the grain boundary with a large angle have been researched. 

## 2. Experimental Methods

### 2.1. Process Stages

The location of carbide solidification was detected by analyzing the solidification sequence of CM247LC. The phase transformation process of the CM247LC superalloy during solidification is as follows: When a superalloy material is cooled below its melting point, the primary solid solution emerges first, followed by the formation of dendrites. During the solidification of superalloy materials, primary carbides (MC) are precipitated from the solution. At this point, primary carbides (MC) and matrix dendrites form simultaneously. After crystallization, the liquid solution gradually precipitates while producing γ/γ′-eutectic cells. Additionally, the γ′ particles precipitate in the dendrite [12,13].

The following explains the function of related alloys in the solidification process:

Titanium (Ti) can improve the alloy’s resistance to hot corrosion, but it is detrimental to its oxidation resistance and casting performance. In addition, an increase in Ti makes the eutectic difficult to dissolve, which makes it more challenging to treat the solution.

Tantalum (Ta) can raise the solution temperature and strength of the γ’ phase during clustering at the γ’ phase, hence effectively improving the alloy’s hot corrosion and oxidation resistance.

Niobium (Nb) can increase the γ’ phase’s high-temperature strength and delay the γ’ phase’s growth, but it could reduce the alloy’s resistance to hot corrosion and oxidation.

Chromium (Cr) primarily occurs as a solid solution in the matrix and creates negligible carbides. Its primary role is to increase the alloy’s hot corrosion and oxidation resistance.

Including Molybdenum (Mo) and Tungsten (W) can improve the high-temperature properties of the alloy by increasing the interatomic bonding force, the diffusion activation energy, and attenuating the diffusion rate. During the clustering at the matrix, Mo could make the lattice mismatch more negative and raise the interface dislocation network density, which is advantageous for enhancing the creep property [14].

During the clustering at grain and dendrites boundaries, C and B not only fill the interstitial spaces of these regions but could also reduce the tearing tendency of these regions. Adding C and B achieve these outcomes by reducing diffusion and forming carbide and boride to strengthen the grain and dendrites boundaries.

The solubility of Hf in the γ’ phase is more significant than in the γ phase, allowing it to strengthen the γ’ phase more effectively. Additionally, Hf is a vital carbide-forming element that can impede the precipitation of M23C6 or M6C at grain borders.

### 2.2. Experiment Material

The chemical composition of the superalloy CM247LC used as the experimental material in this study is provided in Table 1.

### 2.3. Experiment Procedures

#### Investment Casting

This experiment was carried out to analyze the influence of the grain boundary element.

The first step in the investment casting process was producing the shell molds. In particular, the grain selector and rod wax models were coupled with other waxes to form an integrated model. The wax model was dipped 12 times in corundum slurries (front coat and back coat) until the shell thickness reached 7 mm. After the shell mold dried, it was dewaxed, and the model was removed using a steam autoclave and additional heating.

**Figure 1:**
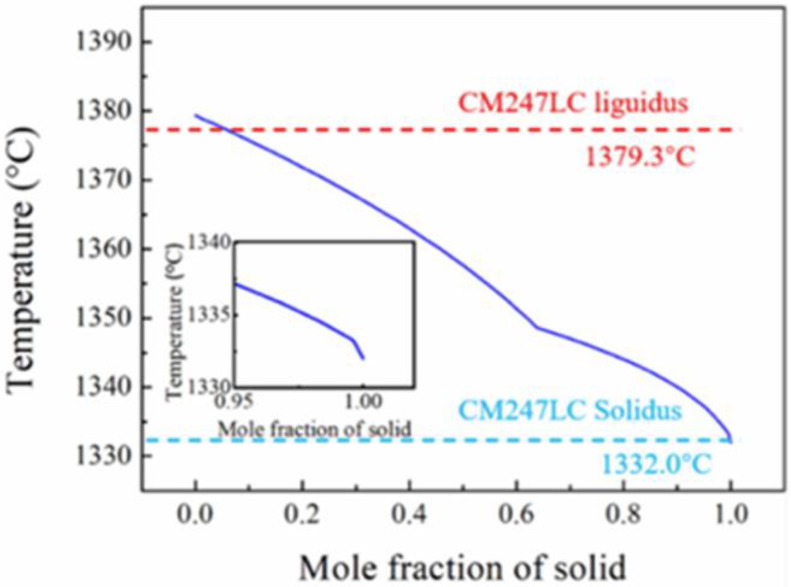
Equilibrium phase fractions between the solidus and liquidus temperatures in experimental alloys: calculated mole fraction of the equilibrium phases for the alloys [15].

**Figure 2:**
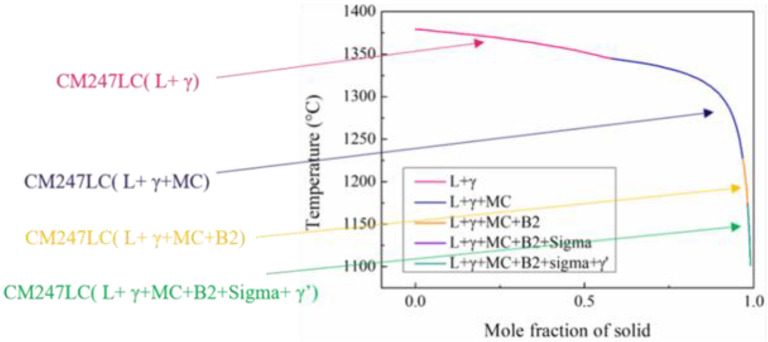
Temperature vs. molar fraction of a solid during the solidification sequence from high to low temperatures for the CM247LC alloy.

The solidification parameters of the alloy are as follows:

In this study, the liquidus and solidus temperatures were calculated and analyzed by Pandat. The liquidus temperature (TL or Tliq) specifies the temperature above which material is completely liquid and the maximum temperature at which crystals can co-exist with the melt in thermodynamic equilibrium. The solidus temperature (TS or Tsol) specifies the temperature below which material completely solidifies and the minimum temperature at which a melt can co-exist with crystals in thermodynamic equilibrium. Figure 1 shows the equilibrium phase fractions between solidus and liquidus temperatures in the experimental alloys. All data were acquired from thermodynamic equilibria calculated using the Pandat software. It shows that the liquidus–solidus temperatures of CM247LC range from 1379.3 to 1332.0 °C. The freezing range (ΔT0) is defined as the temperature range between the liquidus temperature and the solidus temperature of the superalloy. Figure 2 shows the DSC heating curves for the CM247LC superalloy at 10 °C/min heating rates. It can be observed that a large endothermic appeared at 1332.6 °C and peaked at 1378.0 °C, representing the TS and TL, respectively. The DSC result for CM247LC was in agreement with Pandat simulation, which provides a reference for the material properties in the following experiment.

**Figure 3:**
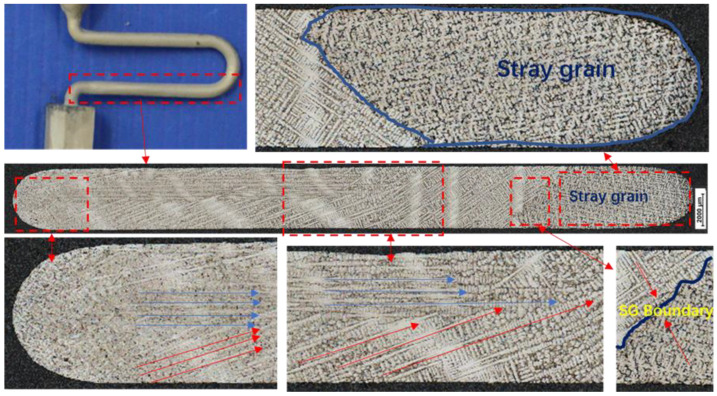
Grain growth direction in the cross selection of the grain selector.

Figure 3 depicts the macrostructure of the inner surface of the ceramic-cored casting with a 15 mm inner diameter and a wall thickness of 2.5 mm. The microstructure of the half cross-section with freckle defect is shown in Figure 3. The increased freckling region is circled as B. Comparing the ceramic core with a 15 mm inner diameter and 0.5 mm wall thickness to the ceramic core with a 2.5 mm wall thickness, the area fraction ratio of the freckling regions on the inner surface of the casting inserted into the ceramic core with a 2.5 mm wall thickness increases by 50% (see Table 2). These findings indicate that the freckle flaw on the inner surface of the casting rises as the wall thickness of the ceramic core increases.

## 3. Results and Discussion

**Figure 4:**
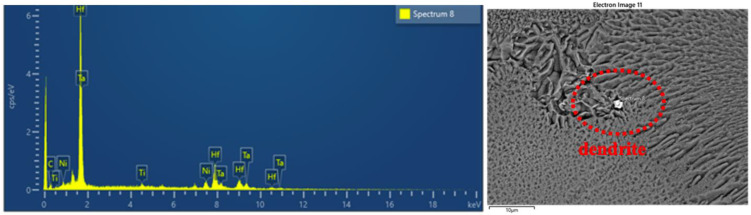
EDS component analysis of Hf distribution of the CM247LC alloy.

The cross-section of the test rod in Figure 4 shows the large angle dendrite growth morphology of the competing dendrite section. Additionally, Table 3 lists the chemical composition of the superalloy in Spectrum 8.

The solidification sequences from high temperatures to low temperatures were as follows:

1. From 1320 °C to 1400 °C, the main component was CM247LC (L + γ).
2. From 1220 °C to 1320 °C, the main component was CM247LC (L + γ + MC).
3. From 1175 °C to 1220 °C, the main component was CM247LC (L + γ + MC + B2).
4. From 1100 °C to 1175 °C, the main component was CM247LC (L + γ + MC + B2 + Sigma + γ’).

The red dotted line represents the intersection of two dendrites at the big angle grain boundary. The green region represents the metallograph of carbide at the grain boundary, where two grains meet, while the black dotted line represents a magnification of the carbide in the center.

The competitive dendrite growth starts from left to right, and the red dotted line marks the entrance of the crystal selection channel (Figure 3). Two dendrites in blue and red enter the transverse crystal selection channel, respectively. Due to the supercooling of the components on the right side of the channel, it first nucleated at the right corner and grew a hetero-grain with an angle of 38° in the 001 direction. The hetero-grain grew to the left and finally encountered the dendrite to form the grain boundary. The grain boundaries are marked in yellow and divided by solid blue lines.

In order to further observe this region, EDS and SEM are used.

As shown in Table 2, the main components of Spectrum 6 are Ni, C, Al, and Cr, with the contents of C reaching 48.66%, Ni reaching 32.90%, and Al reaching 7.45%.

The area at point 6 is the core area for carbide nucleation (MC) as γ/γ′-eutectics. Since the sharp angle of this area reaches 90 degrees, the interface energy of the growth platform at this area is more suitable for nucleation.

During the experiment, Al, Ni, Co, and Cr were not dissolved and were thought to be enriched around MC carbide, which induced the symbiosis of γ′ and γ. As the dendrite solidifies, the pasting zone pushes towards the grain boundary. Then, all the insoluble elements and solute-enriched elements are formed there. The enrichment of elements in grain boundary resulted in the γ/γ′-eutectic reaction around MC carbide.

It can be deduced from the outcomes of EDS and SEM analysis that flocs were formed at the final solidified place at the grain boundary. The EDS component analysis showed that the Hf content reached 29.46% and the carbon content reached 55.55%, which are far higher than the contents of the matrix and carbide. As Hf is a highly insoluble element and is resistant to high temperatures, it can be inferred that the final form is the precipitated hafnium carbide, which is difficult to dissolve and is stored at the grain boundary in the shape of fine floccule.

Hf can lower the temperature of the liquidus and the final solidified region. In other words, Hf could intensify the cold shrinkage of the left and right dendrites when the grain boundary is closed. Furthermore, Hf reduces the amount of liquid required by solidification at grain boundaries to maintain the connections among inter-dendrite fluid pools and has a skin effect.

It can be seen from the metallographic of the dendrite that there is no Hf contained in the dendrite stem of the CM247LC alloy. Additionally, it is highly concentrated in the narrow region between dendrites and often exists in the rich phase. Hf reduces the alloy’s liquidus and brings the final solidification temperature down to 1175 °C. Additionally, the precipitation temperature of the primary phase is also observed to be lower. Due to thermal expansion and cold contraction, the dendrites on both sides are further pushed closer to the grain boundary, making the trend of dendrite closure more obvious.

**Figure 5:**
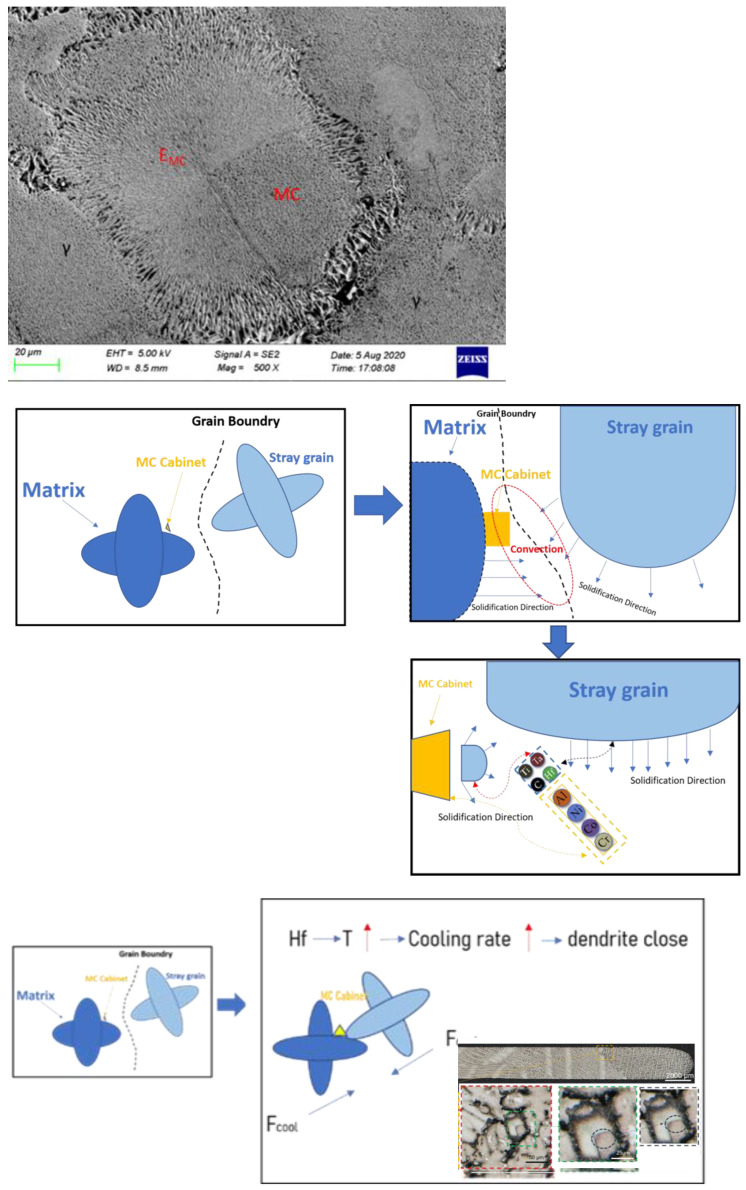
The Hf effect on the growing mode between the MC-type carbide and the eutectic.

Hf has a metamorphic effect on the solidification of the CM247LC alloy, which is mainly reflected in the transformation of carbides from skeleton-like to massive. Since the rich phases are all post-solidified, this metamorphism is not due to the crystallization core created by Hf-rich particles, but it may be due to the reduction in the solidification temperature of the melt and the change in the precipitation sequence of the primary phase of the alloy. Consequently, the former eutectic phase may crystallize freely in the liquid phase or attach to the formed phase, resulting in symbiotic growth. Moreover, the final formed phase has good geometric symmetry, as shown in Figure 5.

It is also observed that the Hf concentration of the solidified melt reaches nearly 30%, which can combine with the elements such as nickel and carbon in the molten pool to form compounds and strengthen the bonding strength of dendrites.

## 4. Conclusions

1. During the solidification process, the alloy material eventually forms γ/γ′-eutectics, in which the chemical composition and crystal structure differ from the primary carbide (MC). It may be that the Al, Ni, Co, and other elements enriched around the primary carbide (MC) provide suitable conditions for the symbiosis of γ/γ′-crystals.
2. The eutectic grows by adsorption on carbides, and it is easier to nucleate and drive the fan growth based on the advantage of the interface energy at the large carbide angle.
3. Finally, the compound of refractory Hf reinforced the dendrite closure benefit and could reduce the crack tendency by controlling the particle size and taking advantage of its effect on the closure of dendrite grain boundary channels.
4. The final region of carbide formation in this experiment is the region where the dendrite is pushed into the paste region and finally solidifies. The performance requirements of carbide on the alloy can be controlled by changing the paste region and solidification sequence.

## Figures and Tables

**Table 1:** The chemical composition of the superalloy CM247LC (wt. %).

Elements	Al	Ti	Cr	Mo	Co	W	Ta	Hf	C	Ni
wt. %	5.49	0.74	8.03	0.5	9.41	9.87	2.9	1.36	0.094	Bal.

**Table 2:** The chemical composition of the superalloy in Spectrum 6.

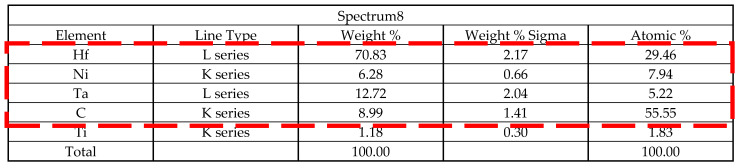

**Table 3:** The chemical composition of the superalloy in Spectrum 8.